# Investigation of gastrointestinal injury‐related biomarkers in dairy cattle with displaced abomasum

**DOI:** 10.1002/vms3.1292

**Published:** 2023-09-30

**Authors:** Merve Ider, Ramazan Yildiz, Amir Naseri, Erdem Gülersoy, Fahrettin Alkan, Mahmut Ok, Alper Erturk, Kadir Sulu, Murat Kaan Durgut

**Affiliations:** ^1^ Faculty of Veterinary Medicine Department of Internal Medicine Selcuk University Konya Turkey; ^2^ Faculty of Veterinary Medicine Department of Internal Medicine Burdur Mehmet Akif Ersoy University Burdur Turkey; ^3^ Faculty of Veterinary Medicine Department of Internal Medicine Harran University Sanlıurfa Turkey; ^4^ Faculty of Veterinary Medicine Department of Surgery Selcuk University Konya Turkey; ^5^ Faculty of Veterinary Medicine Department of Internal Medicine Mustafa Kemal University Hatay Turkey; ^6^ Faculty of Veterinary Medicine Department of Internal Medicine Siirt University Siirt Turkey

**Keywords:** cattle, left displacement of abomasum, leptin, liver‐fatty acid binding proteins, right displacement of abomasum, trefoil factor‐3

## Abstract

**Background:**

Displaced abomasum (DA) is one of the most important metabolic disorders of dairy cattle. In DA, ischaemic damage may occur as a result of impaired perfusion due to abomasal displacement, which may result in gastrointestinal mucosal damage.

**Objective:**

Investigation of gastrointestinal tissue damage in cattle with right displacement of the abomasum (RDA) and left displacement of the abomasum (LDA) using intestinal‐related biomarkers.

**Methods:**

Forty‐eight DA (24 LDA, 24 RDA) and 15 healthy Holstein dairy cows were enrolled between March 2021 and July 2022. Serum biomarkers including gamma‐enteric smooth muscle actin (ACTG‐2), liver‐fatty acid binding proteins (L‐FABP), platelet activating factor (PAF), trefoil factor‐3 (TFF‐3), leptin, claudin‐3 and interleukin‐8 (IL‐8) concentrations were measured from venous blood samples.

**Results:**

L‐FABP concentrations in the LDA group and TFF‐3 concentrations in the RDA group were lower than in the control group. The leptin concentration of the RDA group was higher than that of the other groups. There was a negative correlation between lactate, leptin and IL‐8 concentrations. There was a negative correlation between lactate and TFF‐3, whereas leptin and lactate were positively correlated. Leptin was the more reliable biomarker for discriminating between RDA and LDA cases.

**Conclusion:**

Changes in serum L‐FABP, TFF‐3 and leptin concentrations in cattle with DA may reflect acute intestinal injury and the subsequent repair phase. However, these biomarkers had poor diagnostic performance in discriminating between healthy and cattle with DA, while leptin emerged as the most useful marker in differentiating LDA from RDA cases.

## Graphical abstract

In this study, intestinal injury‐related biomarkers were evaluated in serum samples from dairy cows with displaced abomasum (DA). Serum fatty acid binding proteins, trefoil factor‐3 and leptin concentrations in cattle with DA indicate that intestinal damage develops in DA cases and the repair phase is activated against this damage.

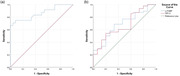

## 1 INTRODUCTION

Displaced abomasum (DA) is one of the most critical problems, especially in high‐yielding dairy cattle, causing economic losses worldwide (Guo et al., 2020; Ok et al., 2002). DA is a multifactorial disorder that occurs as a result of displacement of the abomasum, leading to partial or complete obstruction of the intestinal passage. This causes abnormalities such as ischemia, inflammation, tissue damage and necrosis of the abomasum and intestinal tissue (de Cardoso et al., 2008; Guo et al., 2020).

Haematological biomarkers reflecting metabolic and stress status, tissue damage and inflammatory conditions are useful diagnostic indicators of abdominal disease in dairy cattle (de Cardoso et al., 2008; Guo et al., 2020; Maden et al., 2012; Song et al., 2020). Recently, there has been an increased interest in intestinal‐related biomarkers such as fatty acid binding proteins (FABP), trefoil factor‐3 (TFF‐3), claudin‐3, gamma‐enteric smooth muscle actin (ACTG‐2), leptin, interleukin‐8 (IL‐8) and platelet activating factor (PAF) for the diagnosis and prognosis of gastrointestinal (GI) diseases in preterm infants (Ng et al., 2013), human autopsy samples (Pelsers et al., 2003), premature calves (Yildiz et al., 2019) and calves with atresia coli (Yildiz et al., 2018). These proteins are essential components of the gastrointestinal mucosa and the intestinal barrier. Following injury due to inflammation, hypoxia‐ischemia or bacterial translocation, these intestinal‐related biomarkers are released into the circulation in large quantities from damaged enterocytes (Ng et al., 2013). Although biomarkers related to acute phase response, oxidative stress, lipid and hormonal profile, energy, hepatic and cardiac function have been used to diagnose left displacement of the abomasum (LDA) (Maden et al., 2012; Song et al., 2020), the diagnostic and prognostic value of biomarkers of GI injury has not been investigated in cases of DA.

Therefore, we hypothesized that gastrointestinal‐related biomarkers can be used to predict the severity and prognosis of abomasal displacement in cattle with DA. The aim of this study was to investigate gastrointestinal tissue damage in cattle with abomasal displacement using intestinal‐related biomarkers.

## 2 MATERIALS AND METHODS

The study was conducted with the approval of the Selcuk University Veterinary Faculty Experimental Animal Production and Research Center Ethics Committee with decision number 2021/97.

### 2.1 Animals

#### 2.1.1 Animals with displaced abomasum

Between March 2021 and July 2022, cattle with DA were admitted to the Selcuk University Veterinary Faculty Large Animal Hospital either for diagnosis and/or treatment from small family or medium‐sized dairy farms. The experimental group consisted of 48 (24 with LDA 73 and 24 with RDA) Holstein dairy cows diagnosed with DA within a maximum of 12 h of initial evaluation. All cows with DA were within 2–4 weeks of postpartum, and 80% of the animals were in their third and fourth lactations.

Both general condition and initial clinical signs of RDA cows were similar to those of LDA cows. The history of cows with DA included loss of appetite, decreased rumen motility and rumination and inadequate defecation with a sudden decrease in milk yield. No signs of pain were observed in cows with DA except for two cows with RDA. Rectal examination results were unremarkable. The duration of the symptoms ranged from 2 to 5 days (2–3 and 4–5 for RDA and LDA, respectively). Although these farms have different feed management practices, they do not use a post‐fresh diet (referring to the first 3 days after calving) formulation that contains less than 21% neutral detergent fibre (NDF) from the feed.

#### 2.1.2 Diagnosis of abomasal displacement and exclusion criteria

Cows with DA underwent a complete physical examination (conducted by MI and AE with the same protocol). The diagnosis of DA was based on the presence of a characteristic ping sound on simultaneous auscultation and percussion from both sides of the abdomen and a splashing sound on the abdominal ballotoment. Ultrasonography was performed to confirm the diagnosis of DA and the location of the abomasum (LDA or RDA) using a Mindray DC‐6 Vet, China. In case of suspicion, a Liptak test was performed by inserting a needle into the viscera to remove fluid and measuring the pH of the fluid to confirm its acidic (pH < 4) character (Ok et al., 2002). All cases were confirmed by surgery.

Animals with any postpartum disease (mastitis, metritis, ketosis, retentio secundinariu, pneumonia, foot problems, etc.) other than DA were not included in the study. Animals in all experimental groups were evaluated by vaginal discharge and uterine palpation to exclude metritis and retention. To exclude ketosis, blood concentrations of betahydroxybutyrate (BHB) ≥ 1.2 mmol/L were considered (Oetzel, 2004). In addition, animals with clinical mastitis, pneumonia, foot problems, etc. and those with a history of antibiotic and corticosteroid/nonsteroid administration at least 2 weeks prior to study entry were also excluded.

### 2.2 Healthy cows

The control group consisted of 15 healthy Holstein cows, similar to the DA group, at the same postpartum (2–4 weeks) and lactation (3rd or 4th) period, selected from our faculty farm. Healthy cows were defined as not treated for disease by the farm veterinarian. Control cows underwent a complete physical examination and haematological analysis to ensure that they were healthy prior to inclusion in the study. No abnormalities were found during the physical examination and haematological analysis.

### 2.3 Blood sampling and analyses

Blood samples of 10 mL were collected from all cattle by jugular venipuncture at the time of admission. Plastic syringes containing sodium heparin, tubes containing K_3_EDTA and non‐anticoagulant tubes were used for blood gas, complete blood count (CBC) and serum collection, respectively. Blood gas and CBC were performed within 5–10 min. Blood gas and electrolyte analysis included power of hydrogen (pH), partial pressure of carbon dioxide (pCO_2_), partial pressure of oxygen (pO_2_), potassium (K), sodium (Na), calcium (Ca), chlorine (Cl), glucose (Glu), lactate (Lac), base excess (BE), and bicarbonate (HCO_3_) were measured using an automated blood gas/electrolyte analyser (ABL 90 Flex, Radiometer, USA). Complete blood count parameters including total leukocytes (WBC), lymphocytes (Lym), monocytes (Mon), granulocytes (Gra), erythrocytes (RBC), haematocrit (Hct), haemoglobin (Hb) and platelets (PLT) were measured using an automated cell counter (MS4e® CFE 279, Melet Schlosing Laboratories, France). Blood samples for biomarker analysis were kept at room temperature for 15 min, centrifuged at 2000 × *g* for 10 min, serum extracted and stored at −80°C until the day of analysis.

### 2.4 Biomarker analyses

Serum concentrations of ACTG‐2, L‐FABP, PAF, TFF‐3, leptin, claudin‐3 and IL‐8 were measured using commercially available bovine‐specific ELISA kits (MyBioSource®, San Diego, USA) according to the manufacturer's instructions. The intra‐assay coefficient, inter‐assay coefficient and minimum detection concentration were ≤ 5.5–6.4%, ≤ 6.7–7.9% and 0.1 μg/mL for ACTG‐2; ≤ 15%, ≤ 15% and 0. 05 ng/mL for L‐FABP; ≤ 8%, ≤ 12% and 12 pg/mL for PAF; ≤ 5.9%, ≤ 7.1% and <0.26 ng/mL for TFF‐3; ≤ 8%, ≤ 8% and 0.188 ng/mL for LP; ≤ 8%, ≤ 8% and < 0.188 ng/mL for claudin‐3; ≤ 8%, ≤ 12% and 5 pg/mL for IL‐8.

### 2.5 Statistical analyses

The SPSS 25 statistical programme (IBM Corp®, 2017, Armonk, NY, USA) was used to evaluate the data. The Kolmogorov–Smirnov test was used to determine the normality of the variables and the homogeneity of the variances. Parametric data were expressed as mean ± SD and evaluated by one‐way analysis of variance (ANOVA) followed by post hoc Tukey's test. Non‐parametric data (Na, Cl, Glu, Lac, WBC, Lym, Gra, PLT and intestinal‐related biomarkers) were expressed as median (minimum/maximum) and evaluated using the Kruskal–Wallis test. The Spearman's rank test was used to investigate possible correlations between parameters. Receiver operating characteristic curve (ROC) analysis was used to determine the optimal diagnostic cut‐off values to assess the presence of gastrointestinal damage and to investigate the importance of these biomarkers in differentiating the extent of damage in cases of LDA, RDA and DA. The observed power (Op or post hoc power) was obtained by performing univariate general linear model analysis using the same statistical software. The diagnostic performance of the biomarkers was evaluated using parameters including area under the curve (AUC, >0.600), *p* value (< 0.05), sensitivity, specificity (>70%) and observed power (Op, %). Thresholds for selected ROC parameters were determined in accordance with previous reports to suggest the ability of the test to discriminate between patients with and without disease or condition. For this purpose, it was accepted that an AUC of 0.500 suggests no discrimination, 0.600 to 0.700 suggests poor discrimination, 0.700 to 0.800 is considered acceptable, 0.800 to 0.900 is considered excellent, and more than 0.900 is considered excellent (Hosmer & Lemeshow, 2000). Statistical significance was accepted as *p* < 0.05 for all data.

## 3 RESULTS

### 3.1 Blood gas and CBC

The results of blood gas and CBC analyses are presented in Table 1. In the blood gas analyses, K and Cl levels were lower in the cattle with DA than in the control group, while glucose and lactate concentrations were higher (*p* < 0.05). In the CBC analyses, WBC, Lym, Htc and Hb levels were higher in the LDA and RDA groups, while PLT levels were lower than in the control group (*p* < 0.05).

**TABLE 1:** Blood gas and CBC results for RDA, LDA and controls are expressed as mean ± SD, median and range in parentheses.

Parameters	Control group (*n*:15)	LDA group (*n*:24)	RDA group (*n*:24)
pH	7.40 ± 0.03	7.41 ± 0.06	7.45 ± 0.05
pCO_2_ (mmHg)	43.12 ± 4.21	39.37 ± 4.58	41.54 ± 6.24
pO_2_ (mmHg)	29.25 ± 8.50	30.65 ± 4.63	29.23 ± 4.97
K (mmol/L)	3.88 ± 0.47^a^	3.32 ± 0.62^b^	2.79 ± 0.58^c^
Na (mmol/L)	145^a^ (140–150)	139.5^b^ (121–151)	140.5^a,b^ (140–155)
Ca (mmol/L)	0.98 ± 0.10^a^	0.90 ± 0.08^a^	0.76 ± 0.14^b^
Cl (mmol/L)	104^a^ (102–108)	99.5^b^ (48–108)	92.5^b^ (81–108)
Glu (mg/dL)	50^c^ (42–99)	86^b^ (55–330)	120^a^ (55–224)
Lac (mmol/L)	1^b^ (0.20–2.50)	2.2^a^ (0.50–5.80)	3.4^a^ (0.50–9.90)
BE (mmol/L)	2.48 ± 2.73	1.65 ± 6.65	5.47 ± 6.79
HCO_3_ (mEq/L)	26.84 ± 2.29	25.22 ± 5.25	28.61 ± 5.87
WBC (m/mm^3^)	9.63^b^ (6.29–12.20)	14.16^a^ (3.46–41.42)	19.66^a^ (4.19–59.78)
Lym (m/mm^3^)	3.73^b^ (1.97–6.57)	10.34^a^ (1.67–37.47)	10.96^a^ (1.35–54.57)
Mon (m/mm^3^)	0.45 ± 0.11	0.38 ± 0.14	0.35 ± 0.12
Gra (m/mm^3^)	4.41 (3.22–7.86)	4.28 (1.18–14.65)	5.94 (1–22.32)
RBC (m/mm^3^)	7.46 ± 1.79	7.83 ± 2.04	8.32 ± 1.54
Hct (%)	29.38 ± 4.72^b^	38.56 ± 6.71^a^	37.64 ± 8.40^a^
Hb (g/dL)	9.93 ± 0.96^b^	12.40 ± 1.98^a^	11.96 ± 2.02^a^
PLT (m/mm^3^)	346^a^ (243–1147)	248.5^b^ (135–406)	235.5^b^ (66–729)

*Note*: Different letters (a, b, c) on the same row indicate statistically significant differences (*p* < 0.05).

pCO_2_, partial pressure of carbon dioxide; pO_2_, partial pressure of oxygen; K, potassium; Na, sodium; Ca, calcium; Cl, chloride; Glu, glucose; Lac, lactate; BE, base excess; HCO_3_, bicarbonate; WBC, white blood cell; Lym, lymphocyte; Mon, monocyte; Gra, granulocyte; RBC, red blood cell; Hct, haematocrit; Hb, haemoglobin; PLT, platelets.

### 3.2 Biomarkers

The results of the biomarker analyses are shown in Table 2. The serum L‐FABP concentration of the LDA group was lower than that of the control group (*p* < 0.05). The serum TFF‐3 concentration of the RDA group was lower than that of the control group, while the leptin concentration was found to be higher (*p* < 0.05). In addition, the serum leptin concentration of the RDA group was higher than that of the LDA group (*p* < 0.05).

**TABLE 2:** Biomarker results for RDA, LDA and controls are expressed as mean ± SD, median and range in parentheses.

Parameters	Control group (*n*:15)	LDA group (*n*:24)	RDA group (*n*:24)
ACTG‐2 (μg/mL)	8.56 (6.13–9.90)	8.55 (6.60–12.54)	8.61 (5.37–11.27)
L‐FABP (ng/mL)	78.96^a^ (29.44–173.59)	35.73^b^ (4.50–163.82)	56.00^a,b^ (22.28–163.89)
PAF (pg/mL)	64.58 (27.12–71.65)	60.28 (35.82–132)	64.29 (40.23–196.46)
TFF‐3 (ng/mL)	10.78^a^ (3.86–47.22)	10.61^a,b^ (2.48–31.35)	5.70^b^ (2.31–39.28)
Leptin (ng/mL)	1.04^a^ (0.23–2.99)	0.80^a^ (0.23–3.11)	2.03^b^ (1.04–7.76)
Claudin‐3 (ng/mL)	5.63 (3.31–8.43)	5.63 (1.24–12.34)	5.34 (2.84–17.99)
IL‐8 (pg/mL)	15.89 (6.66–18.62)	17.52 (10.02–98.47)	18.62 (11.06–28.86)

*Note*: Different letters (a, b) on the same row indicate statistically significant differences (*p* < 0.05).

ACTG‐2, intestinal smooth muscle actin; L‐FABP, liver‐fatty acid binding protein; PAF, platelet activating factor; TFF‐3, trefoil factor‐3; IL‐8, interleukin‐8.

### 3.3 Correlations

The results of the correlation analysis are shown in Table 3. There was a positive correlation between K, Ca and TFF‐3 concentrations and a negative correlation between lactate, leptin and IL‐8 concentrations. There was also a negative correlation between Na, lactate, claudin‐3 and IL‐8 concentrations. Ca concentration was positively correlated with TFF‐3, whereas it was negatively correlated with lactate. There was a negative correlation between lactate and TFF‐3, while leptin and lactate were positively correlated. There was also a positive correlation between PAF and TFF‐3 concentrations.

**TABLE 3:** Results of Spearman's correlation analyses between venous blood gas parameters and biomarker concentrations in cattle with DA and healthy cattle.

	K	Na	Ca	Lac	PAF	TFF‐3	Leptin	Claudin‐3	IL‐8
**K**	1	0.157	0.671^**^	–0.432^**^	–0.171	0.393^**^	–0.289^*^	0.128	–0.257^*^
**Na**		1	–0.114	–0.334^**^	–0.196	–0.046	–0.157	–0.273^*^	–0.257^*^
**Ca**			1	‐0.284^*^	–0.029	0.349^**^	–0.238	–0.001	–0.113
**Lac**				1	0.186	–0.252^*^	0.354^**^	0.095	0.126
**PAF**					1	0.283^*^	0.179	–0.015	0.244
**TFF‐3**						1	–0.080	0.016	–0.038
**Leptin**							1	0.113	0.103
**Claudin‐3**								1	–0.040
**IL‐8**									1

Lac, lactate; PAF, platelet activating factor; TFF‐3, trefoil factor‐3; IL‐8, interleukin‐8.

*p < 0.05.

**p < 0.01.

### 3.4 ROC analysis

**FIGURE 1:**
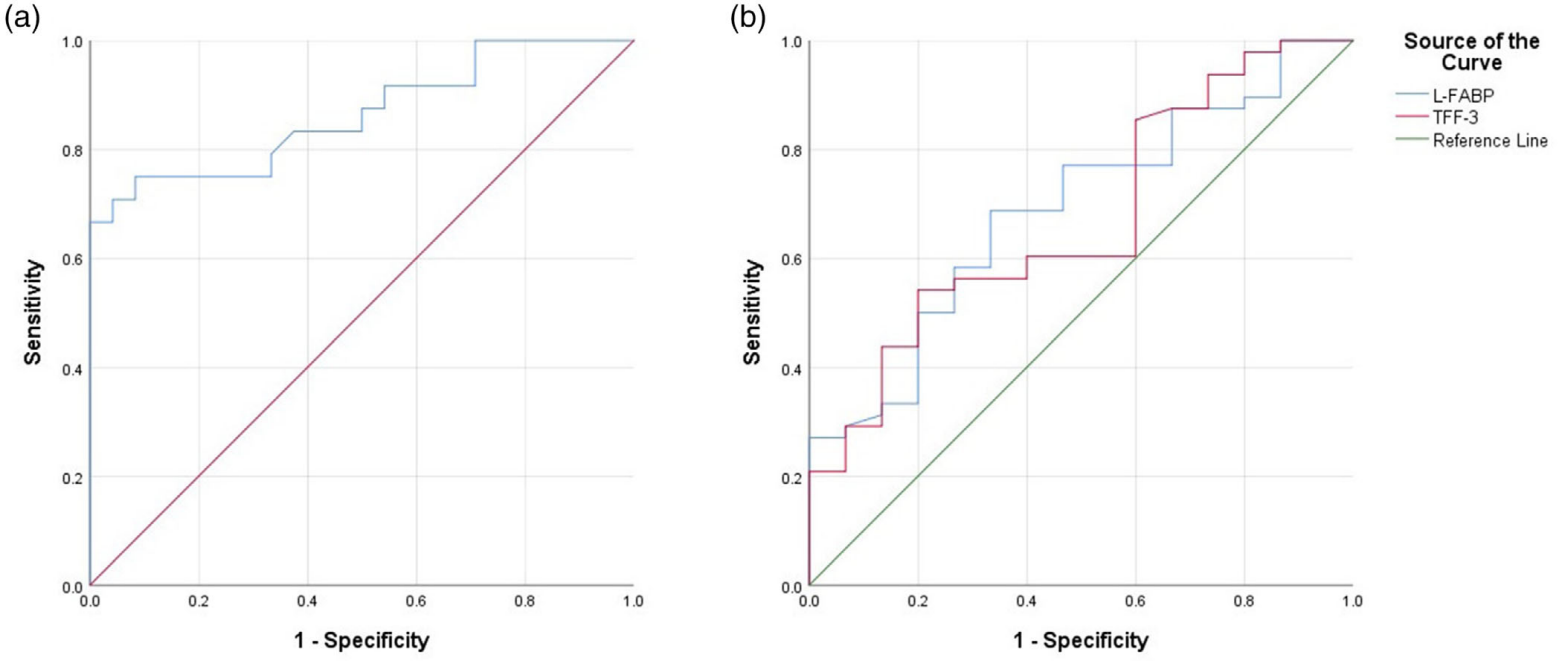
(a) Receiver operating characteristic (ROC) analysis to discriminate LDA from RDA cases based on serum leptin concentrations. (b) Receiver operating characteristic (ROC) analysis to discriminate DA cases from control cattle based on serum L‐FABP and TFF‐3 concentrations.

The results of the ROC analysis performed showed that leptin concentration at the cut‐off point of 1.01 ng/mL, area under the curve (AUC) 0.864 (95% confidence interval (CI) 0.758–0.970; *p* < 0.001), with 100% sensitivity and 66.7% specificity was the best biomarker for discriminating between cattle with LDA and RDA (Table 4, Figure 1a). Furthermore, L‐FABP concentration at the cut‐off point of 45.73 ng/mL, area under the curve (AUC) 0.688 (95% confidence interval (CI) 0.543–0.834; *p* < 0.001), with 80% sensitivity and 50% specificity and TFF‐3 concentration at the cut‐off point of 8. 02 ng/mL, area under the curve (AUC) 0.672 (95% confidence interval (CI) 0.521–0.822; *p* < 0.001), with 80% sensitivity and 54.2% specificity, have a poor diagnostic discrimination performance for differentiating between healthy cattle and cattle with DA (Table 5, Figure 1b).

**TABLE 4:** ROC analysis results for discrimination between LDA and RDA cases.

				Asymp. %95 CI							
Parameters	AUC	Std. error	Asymp. Sig	Lower Bound	Upper Bound	Cut‐off	Sensitivity %	Specificity %	PPV	NPV	Op %
**ACTG‐2**	0.462	0.088	0.650	0.289	0.634	7.45	62.5	16.7	44.4	42.8	72.1
**L‐FABP**	0.649	0.081	0.076	0.490	0.809	26.96	91.7	41.7	40.5	1	74.5
**PAF**	0.606	0.082	0.208	0.444	0.767	58.49	70.8	45.6	37	58.3	47.8
**TFF‐3**	0.328	0.079	0.041	0.174	0.483	6.2	50	33.3	52	85.7	85.9
**Leptin**	0.864	0.054	0.000	0.758	0.970	1.01	100	66.7	25.8	12.5	93.5
**Claudin‐3**	0.497	0.085	0.975	0.330	0.665	4.84	58.3	37.5	44	71.4	48.2
**IL‐8**	0.576	0.086	0.364	0.408	0.745	16.49	79.2	50	26.9	38.4	63.9

ACTG‐2, intestinal smooth muscle actin; L‐FABP, liver‐fatty acid binding protein; PAF, platelet activating factor; TFF‐3, trefoil factor‐3; IL‐8, interleukin‐8; PPV, positive predictive value; NPV, negative predictive value.

**TABLE 5:** ROC analysis results for discrimination between DA cases and control group.

				Asymp. %95 CI							
Parameters	AUC	Std. error	Asymp. Sig	Lower Bound	Upper Bound	Cut‐off	Sensitivity %	Specificity %	PPV	NPV	Op %
**ACTG‐2**	0.492	0.074	0.929	0.348	0.637	7.8	80	33.3	72.7	42.8	84.2
**L‐FABP**	0.688	0.074	0.029	0.543	0.834	45.73	80	50	66.6	11.1	94.3
**PAF**	0.481	0.079	0.821	0.325	0.636	52.8	86.7	33.3	94.1	10.3	69.4
**TFF‐3**	0.672	0.077	0.046	0.521	0.822	8.02	80	54.2	64.7	10.3	75.9
**Leptin**	0.303	0.080	0.022	0.147	0.460	1.01	53.3	33.3	80	28	75.7
**Claudin‐3**	0.522	0.071	0.796	0.383	0.661	4.4	80	41.5	71.4	14.2	59.8
**IL‐8**	0.365	0.074	0.118	0.221	0.510	14.34	73.3	29.2	76	23.5	64.4

ACTG‐2, intestinal smooth muscle actin; L‐FABP, liver‐fatty acid binding protein; PAF, platelet activating factor; TFF‐3, trefoil factor‐3; IL‐8, interleukin‐8; PPV, positive predictive value; NPV, negative predictive value.

## 4 DISCUSSION

In this study, intestinal injury‐related biomarkers such as ACTG‐2, L‐FABP, PAF, TFF‐3, leptin, claudin‐3 and IL‐8 were evaluated in serum samples from dairy cows with DA. It was found that serum concentrations of leptin, L‐FABP and TFF‐3 could provide useful information in the evaluation of clinical‐pathological changes in DA.

Because there are limited studies evaluating intestinal injury in veterinary medicine, the results of this study are discussed in the context of studies conducted in both human and veterinary medicine. Biomarkers related to gastrointestinal injury play a role in the presence of damage, protection and/or repair processes of the gastrointestinal tract (Fernandez‐Riejos et al., 2010; Ok et al., 2020). Plasma concentrations of both I‐FABP and L‐FABP were found to increase together in bowel‐related disorders, whereas only L‐FABP increased in patients with liver injury (Pelsers et al. 2003). L‐FABP has been found to be an important endogenous cytoprotectant that minimises oxidative damage to hepatocytes and interferes with ischemia‐reperfusion and other liver injuries (Mukai et al., 2017; Wang et al., 2015). It has also been reported that hepatic triglyceride accumulation and fatty acid uptake are reduced in L‐FABP‐free mice (Newberry et al., 2003). Contrary to what we expected, the serum L‐FABP concentration of the LDA group was found to be lower than that of the control group. It had a sensitivity of 80% and a specificity of 50% for differentiating between healthy cattle and cattle with DA. As DA cases are mainly associated with fatty liver (Maden et al., 2012; Song et al., 2020), the low L‐FABP concentration in cattle with LDA may be related to the hepatic protective effect of L‐FABP (Mukai et al., 2017; Newberry et al., 2003; Wang et al., 2015). Hypoperfusion and intestinal damage, on the one hand, and fatty liver disease and a decrease in the protective mechanism due to fatty acid accumulation, on the other hand, in combination may lead to the statistically insignificant findings in RDA cases. This may explain the lack of significant changes in L‐FABP concentrations in RDA cases.

Trefoil factor‐3 is a compact peptide involved in essential functions such as mucosal protection and cell proliferation (Emami et al., 2004). In rats with colitis, mucosal expression of TFF‐3 in the distal colon was found to decrease in the acute phase and increase in the healing phase and to play an active role in repairing mucosal damage (Itoh et al., 1996). Another study in mice found that TFF‐3 expression was reduced and completely depleted in severe intestinal injury. In the same study, TFF‐3 expression from intestinal goblet cells started during the repair phase and its concentrations gradually increased (Xian et al., 1999). In addition, TFF‐3 concentrations were found to be useful and reliable for determining intestinal epithelial damage in bovine studies (Ok et al., 2020; Yildiz et al., 2018; Yildiz et al., 2019). In the present study, the serum TFF‐3 concentration of the RDA group was found to be lower than that of the other groups, and a sensitivity of 80% and a specificity of 54.2% showed poor diagnostic discriminatory performance between healthy cattle and cattle with DA. Contrary to our expectations, these results may also be explained by the fact that the low serum TFF‐3 concentration of the RDA group was due to the acute injury phase of the cattle and the severity of the general clinical signs in cows with RDA compared to cows with LDA (Rohn et al., 2004).

In addition, serum TFF‐3 was positively correlated with K, Ca and PAF and negatively correlated with lactate. Low circulating concentrations of lactate are found in healthy individuals, but when the intestinal barrier is injured/dysfunctional, these concentrations increase as a result of increased translocation across the intestinal mucosa (Li et al., 2017). Thus, the repair phase of intestinal injury may explain the negative correlation between TFF‐3 and lactate. A previous study reported that TFF‐3 expression is Ca dependent and its release may change at different pH levels (Madsen et al., 2013). It has also been found that TFF‐3 protects the intestinal epithelium from PAF‐induced degradation (Xu et al., 2011). Metabolic and acid‐base changes in DA may also be responsible for the current correlations found with TFF‐3 concentrations (Eisenhut, 2006; Garcia‐Hernandez et al., 2017).

Leptin is a pluripotent peptide hormone mainly produced by adipocytes and other tissues such as the stomach and intestines (Song et al., 2020). It was determined that leptin treatment significantly reduced tissue damage in rats with ischemia‐reperfusion injury (Deng et al., 2012; Hacioglu et al., 2005). Similarly, it was reported that leptin concentrations increased significantly in calves with enteritis, and it was concluded that this increase might be related to the role of leptin in repairing the damaged intestinal barrier and mucosal defence by stimulating the release of growth hormone (Pelsers et al., 2003). We expected that leptin, which is produced by the gastric mucosa and is found in the proximal lumen of the small intestine, would increase its concentration as a result of the displacement of the abomasum. In the present study, the serum leptin concentration of the RDA group was significantly higher than the other groups and with a sensitivity of 100% and a specificity of 66.7% was the best biomarker for discriminating between cattle with LDA and RDA. According to these results, the high serum leptin concentration in the RDA group was interpreted as an indicator of intestinal damage and the repair process of the damaged intestinal barrier and proliferation, which may indicate a better prognosis as they are indicative of resolution of ischemia, inflammation and necrosis (Maden et al., 2012; Pelsers et al., 2003) in cases of RDA.

On the other hand, leptin has pleiotropic functions such as energy homeostasis and the ability to regulate inflammation (de Candia et al., 2021). Leptin secretion causes an increase in blood glucose and lactate concentration (Mueller et al., 2000). It has also been reported that leptin production triggers aldosterone secretion and may lead to secondary hypokalaemia (Huby et al., 2015). In the present study, consistent with the pleotropic effect of leptin (de Candia et al., 2021), a negative correlation was found between serum leptin and K and a positive correlation with lactate concentrations. The correlation between lactate and K concentrations can be interpreted as a result of metabolic effects (Huby et al., 2015; Mueller et al., 2000) of leptin.

During the inflammatory response and production of inflammatory mediators (tumour necrosis factor, gamma interferon, interleukins, leukotrienes and bradykinin) alterations occur in the functions of membranous ion transport system (Ng et al., 2013). Claudin‐3, an important tight junction protein of intestinal epithelium and endothelium, is also effective in ion transport (Ng et al., 2013; Wang et al., 2015). In the present study, there were negative correlations between claudin‐3 and Na. In addition, IL‐8 was negatively associated with K and Na levels. These findings have been linked to changes in ion transport caused by the inflammatory response in DA (Ng et al., 2013; Wang et al., 2015).

Although we attempted to match control and ill animals for factors such as age, diet, parity and duration of illness, these animals were admitted to our hospital from different farms, which may have affected biomarker concentrations. A small study population resulted from this standardisation effort. In addition, the lack of histopathological confirmation of intestinal damage with biomarkers is one of the limitations of this study. All these issues deserve to be addressed in further studies.

## 5 CONCLUSION

In conclusion, alterations in serum liver‐fatty acid binding proteins, trefoil factor‐3 and leptin concentrations in cattle with DA indicate that intestinal damage develops in DA cases and the repair phase is activated against this damage. However, these biomarkers had poor diagnostic performance in discriminating between healthy and cattle with DA, while leptin emerged as the most useful marker in differentiating LDA from RDA cases.

## AUTHOR CONTRIBUTIONS

Conceptualisation: Ider M, Ok M, Yildiz R. Data curation: Alkan F, Erturk A, Durgut MK, Sulu K. Formal analysis: Ider M, Naseri A, Erturk A, Durgut MK, Yildiz R. Funding acquisition: Ider M, Erturk A, Durgut MK. Investigation: Ider M, Ok M, Erturk A, Yildiz R, Gülersoy E. Methodology: Ider M, Erturk A, Durgut MK. Project administration: Ider M, Erturk A, Durgut MK. Resources: Ider M, Ok M, Yildiz R. Software: Ider M, Naseri A, Ok M, Erturk A, Gülersoy E. Supervision: Ider M, Ok M, Yildiz R. Validation: Alkan F, Erturk A, Durgut MK, Sulu K. Visualisation: Erturk A, Durgut MK. Writing – original draft: Ider M, Naseri A, Erturk A, Durgut MK. Writing – review & editing: Ider M, Naseri A, Ok M, Erturk A, Gülersoy E.

## CONFLICT OF INTEREST STATEMENT

The authors declare no conflicts of interest.

## FUNDING INFORMATION

None.

## ETHICS STATEMENT

The study was conducted with the approval of the Selcuk University Veterinary Faculty Experimental Animal Production and Research Center Ethics Committee with decision number 2021/97.

## Data Availability

The data that support the findings of this study are openly available in [repository name e.g. “figshare”] at https://doi.org/10.1002/vms3.1292], reference number [reference number].
